# Design of an Internal/External Bicontinuous Conductive Network for High-Performance Asymmetrical Supercapacitors

**DOI:** 10.3390/molecules27238168

**Published:** 2022-11-23

**Authors:** Anran Shi, Xiumei Song, Lei Wei, Huiyuan Ma, Haijun Pang, Weiwei Li, Xiaowei Liu, Lichao Tan

**Affiliations:** 1 School of Materials Science and Chemical Engineering, Harbin University of Science and Technology, Harbin 150040, China; 2 Institute of Carbon Neutrality, Zhejiang Wanli University, Ningbo 315100, China; 3 Chilwee Power Co., Ltd., No. 18 Chengnan Road, Huaxi Industrial Zone, Changxing 313100, China; 4 State Key Laboratory of Clean Energy Utilization, School of Materials Science and Engineering, Zhejiang University, Hangzhou 310027, China

**Keywords:** Ni-based bimetallic sulfide, core-shell structure, bicontinuous conductive network, polypyrrole coating, asymmetric supercapacitors

## Abstract

High-energy density supercapacitors have attracted extensive attention due to their electrode structure design. A synergistic effect related to core–shell structure can improve the energy storage capacity and power density of electrode materials. The Ni-foam (NF) substrate coupled with polypyrrole (PPy) conductive coating can serve as an internal/external bicontinuous conductive network. In this work, the distinctive PPy@FeNi_2_S_4_@NF and PPy@NiCo_2_S_4_@NF materials were prepared by a simple two-step hydrothermal synthesis with a subsequent in situ polymerization method. PPy@FeNi_2_S_4_@NF and PPy@NiCo_2_S_4_@NF could deliver ultrahigh specific capacitances of 3870.3 and 5771.4 F·g^−1^ at 1 A·g^−1^ and marvelous cycling capability performances of 81.39% and 93.02% after 5000 cycles. The asymmetric supercapacitors composed of the prepared materials provided a high-energy density of over 47.2 Wh·kg^−1^ at 699.9 W·kg^−1^ power density and 67.11 Wh·kg^−1^ at 800 W·kg^−1^ power density. Therefore, the self-assembled core–shell structure can effectively improve the electrochemical performance and will have an effective service in advanced energy-storage devices.

## 1. Introduction

In recent years, the ever-increasing amount of energy consumption has posed a serious challenge to humans [1,2]. Supercapacitors, also called electrochemical capacitors, have been paid more and more attention because of their high power density, fast charge–discharge process, and long lifetime [3,4,5]. In the present study, these pseudocapacitive materials still suffer from low conductivity and large volume change during the charge–discharge process, which is incapable of the requirements of new energy storage devices [6,7,8]. Meanwhile, the inherent defect coming from the electrode materials is also an important influence factor for regrettable electrochemical behavior. To further improve the electrochemical performance of pseudocapacitive materials, several approaches such as nanospheres [9,10], nanosheets [11,12,13], nanowires [14,15,16] shorten the transmission path of ions, thus accelerating ion diffusion, and coated conductive material such as carbon nanotubes [17,18,19] and graphene [20,21] improve the electronic conductivity. Furthermore, heterostructure composites with nanostructures fabricated by assembling different electrode materials together apparently supply enhanced electrochemical performance as desired due to the synergistic effects of different materials [22,23].

Currently, many transition metal oxides (TMOs) and transmission metal sulfides (TMSs) can be promising options, which are namely NiCo_2_O_4_ [24], MnCo_2_O_4_ [25], MnCo_2_S_4_ [26], and MoS_2_ [27]. Among them, the TMSs have been considered and recommended as supercapacitors materials because of their better electrical conductivity and diversified redox reaction when compared to TMOs. The alternate of sulfur with oxygen can obtain a much easier flexible architecture on account of the electronegativity of oxygen being more than that of sulfur. Because of the flexible chemical bonds and narrow band gap, which provide better electron transit within the structure and are suitable for the enhancement of electrochemical activities, the architecture is prevented from collapsing. Nickel-based monometallic sulfurated electrode materials are difficult to use to achieve satisfactory results in terms of electrical conductivity as well as electrochemical performance, and their wide forbidden bandwidth limits the number of electron carriers and their utilization efficiency [28,29,30,31]. The nickel-based bimetallic composites may produce complementary and synergistic effects on the intrinsic characteristics and obtain more beneficial electrochemistry properties.

With the above analysis, we prepared PPy@FeNi_2_S_4_@NF and PPy@NiCo_2_S_4_@NF materials that display superior electrochemical performance by using a two-step hydrothermal and in situ polymerization method. Specifically, the as-fabricated materials are owed to the unique core–shell structure with more electroactive sites, stable structure and synergistic effects. Profiting from the features of special structure, PPy@FeNi_2_S_4_@NF presented a specific capacitance of 3908.5 F·g^−1^ at 1 A·g^−1^ and good cycling stability with a capacity retention of 81.39% after 5000 cycles at 10 A·g^−1^. PPy@NiCo_2_S_4_@NF showed a specific capacitance of 5142.8 F·g^−1^ at 1 A·g^−1^ and a remarkable cycling stability of about 93.02% of the initial specific capacitance after 5000 cycles at 10 A·g^−1^. Furthermore, an asymmetric supercapacitor was assembled with PPy@FeNi_2_S_4_@NF and PPy@NiCo_2_S_4_@NF as the positive electrodes and activated carbon (AC) as the negative electrode, which showed a high energy density of 47.2 Wh·kg^−1^ at a power density of 699.9 W·kg^−1^ and 67.11 Wh·kg^−1^ at a power density of 800 W·kg^−1^. The above results imply that PPy@FeNi_2_S_4_@NF and PPy@NiCo_2_S_4_@NF are promising electrode materials in supercapacitors for practical application.

## 2. Results and Discussion

**Figure 1:**
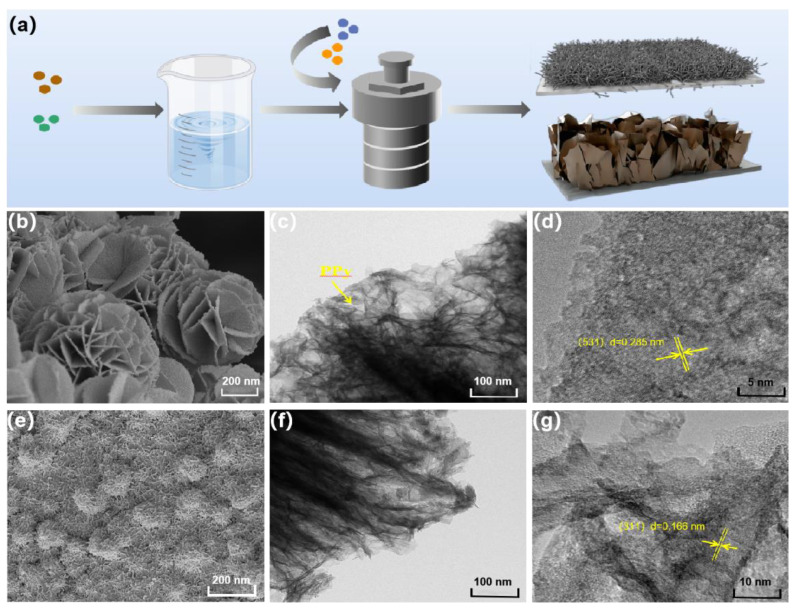
(**a**) Schematic illustrating the fabrication route of PPy@FeNi_2_S_4_@NF and PPy@NiCo_2_S_4_@NF. SEM images of (**b**) PPy@FeNi_2_S_4_, (**e**) PPy@NiCo_2_S_4_@NF. (**c**,**f**) TEM images of PPy@FeNi_2_S_4_@NF and PPy@NiCo_2_S_4_@NF. (**d**,**g**) High-resolution TEM image of PPy@FeNi_2_S_4_@NF and PPy@NiCo_2_S_4_@NF.

The synthesis process of PPy@FeNi_2_S_4_@NF and PPy@NiCo_2_S_4_@NF is shown in Figure 1a. FeNi_2_S_4_ nanosheets and the NiCo_2_S_4_ nanowire array were prepared by a hydrothermal method based on foamed nickel material, and PPy was coated and synthesized by an in situ polymerization method to form nano-materials with a core–shell structure. The SEM images of Figure 1b,e show that PPy@FeNi_2_S_4_@NF is composed of ultrathin nanosheets and PPy@NiCo_2_S_4_@NF are nanowires with a radius of 20–30 nm. Due to the independent 3D interconnection structure, NF was selected as the substrate, which can be directly used as a current collector. The SEM images of PPy@FeNi_2_S_4_@NF and PPy@NiCo_2_S_4_@NF composites at different reaction times are shown in Figures S1 and S2. Moreover, the TEM, as shown in Figure 1c,f, further confirms the ultrathin nanosheets of PPy@FeNi_2_S_4_@NF and nanowires of PPy@NiCo_2_S_4_@NF. Simultaneously, it can be seen that the surface of the materials is coated by PPy, and the surface of the materials also presents the unique wrinkled texture of PPy. The core–shell structure material can store more charges, and the nano-array inside the shell forms a uniformly dispersed three-dimensional structure, which not only increases the specific surface area and provides a high specific capacity but also provides a nano-channel for fast electron transmission and improves its conductivity. The HRTEM image of PPy@FeNi_2_S_4_@NF nanosheets and PPy@NiCo_2_S_4_@NF nanowires exhibit clear lattice fringes with interplanar spacing of 0.285 and 0.166 nm, corresponding to the (531) plane and (311) plane in Figure 1d,g.

**Figure 2:**
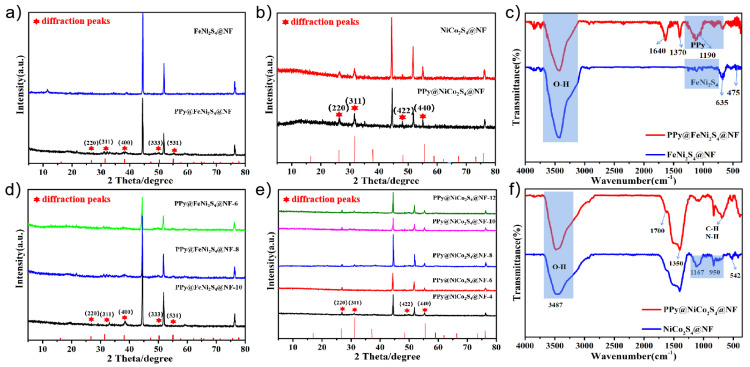
(**a**) XRD patterns of FeNi_2_S_4_@NF and PPy@FeNi_2_S_4_@NF, (**b**) XRD patterns of NiCo_2_S_4_@NF and PPy@NiCo_2_S_4_@NF, (**d**,**e**) XRD patterns of PPy@FeNi_2_S_4_@NF and PPy@NiCo_2_S_4_@NF at different reaction times, (**c**) FTIR spectra of FeNi_2_S_4_@NF and PPy@FeNi_2_S_4_@NF, and (**f**) FTIR spectra of NiCo_2_S_4_@NF and PPy@NiCo_2_S_4_@NF.

The X-ray diffraction (XRD) patterns of PPy@FeNi_2_S_4_@NF and PPy@NiCo_2_S_4_@NF are shown in Figure 2a,b. The diffraction peak of the prepared composite is consistent with the standard peak of FeNi_2_S_4_ and NiCo_2_S_4_. In addition to the diffraction peaks of nickel foam, a series of diffraction peaks belonging to NiFe_2_S_4_ at 27.5°, 32.7°, 38.3°, 50.9° and 57.2° correspond to (220), (311), (400), (333) and (531) crystal planes and a series of diffraction peaks belonging to NiCo_2_S_4_ at 26.4°, 31.8°, 47.8° and 55.6° correspond to (220), (311), (422), (333) and (440) [32,33,34]. Figure 2d,e show the XRD curves of composite material PPy@FeNi_2_S_4_@NF and PPy@NiCo_2_S_4_@NF with different reaction times. It can be seen that the number and position of the peaks are inconspicuously changed [35,36].

Further investigation was conducted through the FTIR analysis to detect that the PPy material was successfully compounded onto the surface. As the FTIR spectra show in Figure 2c, the band at 1190 cm^−1^ is assigned to the C−N stretching vibration between the two pyrrole rings. The characteristic peaks at 1370 cm^−1^ are caused by symmetric and antisymmetric vibrations of the pyrrole ring [18,37]. The band at 1640 cm^−1^ is caused by C=C stretch vibration. In addition, the FTIR spectra of PPy@NiCo_2_S_4_@NF in Figure 2f also matched well with the characteristic peak of PPy. At the peak of 1350 cm^−1^, it can be seen that the peak intensity of the final product is obviously higher than that of the intermediate product, which is related to the cyclic stretching mode of PPy [38,39].

**Figure 3:**
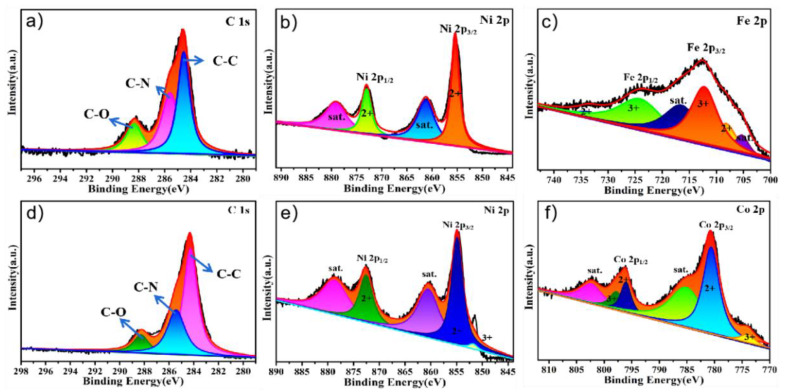
XPS survey scan of the PPy@FeNi_2_S_4_@NF: high-resolution XPS spectra of (**a**) C 1s, (**b**) Ni 2p and (**c**) Fe 2p. XPS survey scan of the PPy@NiCo_2_S_4_@NF: high-resolution XPS spectra of (**d**) C 1s, (**e**) Ni 2p and (**f**) Co 2p.

The XPS spectra of PPy@FeNi_2_S_4_@NF and Ppy@NiCo_2_S_4_@NF were performed to corroborate the successful synthesis of the composite materials. The survey spectra, as shown in Figure S3a, indicate the existence of C, O, N, Ni, Fe and S elements on the surface of PPy@FeNi_2_S_4_@NF. There are three peaks at 288.20, 286.15 and 284.73 eV, which belong to the peaks of C−O, C−N and C−C bonds, respectively. In Figure 3b, the XPS spectrum of Ni 2p shows four peaks with different peaks. The peak at 872.82 eV belongs to the low coordination orbit of Ni, while the peak at 855.30 eV corresponds to the high coordination orbit of Ni [40,41]. Figure 3c shows the two peaks corresponding to Fe^2+^ at 734.67 and 704.63 eV, and the peaks at 724.17 and 714.38 eV correspond to Fe^3+^ [42,43]. The XPS spectrum of N 1s shows two peaks at 399.7 and 398.3 eV in Figure S2, which are attributed to pyrrole N and pyridine N [44].

The survey spectra, as shown in Figure S3d, indicate the existence of C, O, N, Ni, Co and S elements on the surface of the as−prepared composite PPy@NiCo_2_S_4_@NF. There are three peaks at 288.0, 285.4 and 284.3 eV [45,46], which belong to the peaks of C−O, C−N and C−C bond, respectively, in Figure 3d. It can be seen in Figure 3e that there are two peaks corresponding to Ni^2+^ at 872.27 and 860.15 eV, and the peak at 854.75 eV corresponds to Ni^3+^ [47,48]. The XPS spectrum of Co 2p shows six different peaks in Figure 3f. The peaks at 802.05 and 775.76 eV belong to Co^3+^, while the peaks at 796.34 and 780.49 eV correspond to Co^2+^ [49,50].

**Figure 4:**
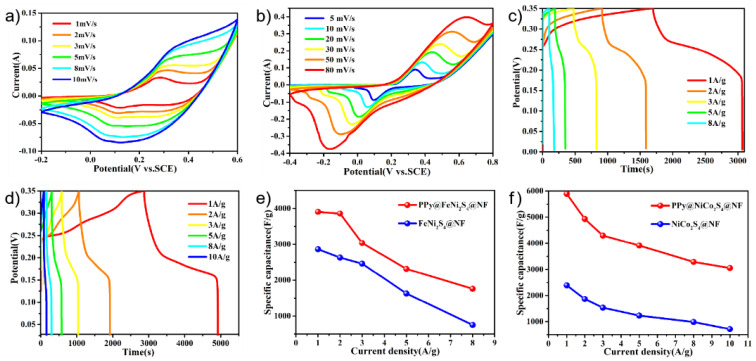
(**a**) CV curves of the PPy@FeNi_2_S_4_@NF electrode at various sweeping rates. (**b**) CV curves of the PPy@NiCo_2_S_4_@NF electrode at various sweeping rates. (**c**) Specific capacitance of PPy@FeNi_2_S_4_@NF electrode at different current densities. (**d**) Specific capacitance of PPy@NiCo_2_S_4_@NF electrode at different current densities. (**e**) Specific capacitance of as-prepared electrodes at different current densities of the PPy@FeNi_2_S_4_@NF and FeNi_2_S_4_@NF electrode. (**f**) Specific capacitance of as-prepared electrodes at different current densities of the PPy@NiCo_2_S_4_@NF and NiCo_2_S_4_@NF electrode.

The electrochemical performances of the PPy@FeNi_2_S_4_@NF and PPy@NiCo_2_S_4_@NF materials were tested and used as working electrodes for the supercapacitors in the three-electrode system. Figure 4a,b shows the cyclic voltammetry of PPy@FeNi_2_S_4_@NF-6 and PPy@NiCo_2_S_4_@NF at different scan rates over a constant voltage range. With an increase in scanning rate, the oxidation–reduction process speeds up. However, the basic shape of the curve remains unchanged, which proves that the material has excellent rate performance [51,52]. By comparison, Figure S4 proves that PPy is compounded with the material, and the specific capacity of the electrode is improved. As shown in Figure 4c, the PPy@FeNi_2_S_4_@NF electrode has a specific capacitance of 3870.3 F·g^−1^ at 1 A·g^−1^, which is much higher than that of the FeNi_2_S_4_@NF electrode material in Figure S5. In Figure 4d, the PPy@NiCo_2_S_4_@NF electrode has a specific capacitance of 5771.4 F·g^−1^ at a current density of 1 A·g^−1^, which is much higher than that of the NiCo_2_S_4_@NF electrode material, as shown in Figure S6. As shown in Figure S7, the specific capacity of PPy@FeNi_2_S_4_@NF-x and PPy@ NiCo_2_S_4_@NF-x reached the maximum when the reaction time was 6 h. The composite of PPy provides an extra specific capacity of an electric double layer, and this coating structure can store more ions inside the active material, thus absorbing more charges [53].

According to Figure 4e,f, the specific capacitances of FeNi_2_S_4_@NF and NiCo_2_S_4_@NF are lower than the PPy@FeNi_2_S_4_@NF and PPy@NiCo_2_S_4_@NF material at any current densities. At low current density, it can provide sufficient time for ion transmission. At high current density, it shows a lower specific capacitance due to the influence of ion transmission rate, and it shows that the electrode material after PPy coating has excellent rate performance. In short, the conductive polymer layer and the conductive substrate (NF) can generally produce a novel internal/external conductive structure by coating a certain amount of PPy. This dual conductive structure could greatly promote the electron transport efficiency, which may further enhance the rate performance [52,54].

**Figure 5:**
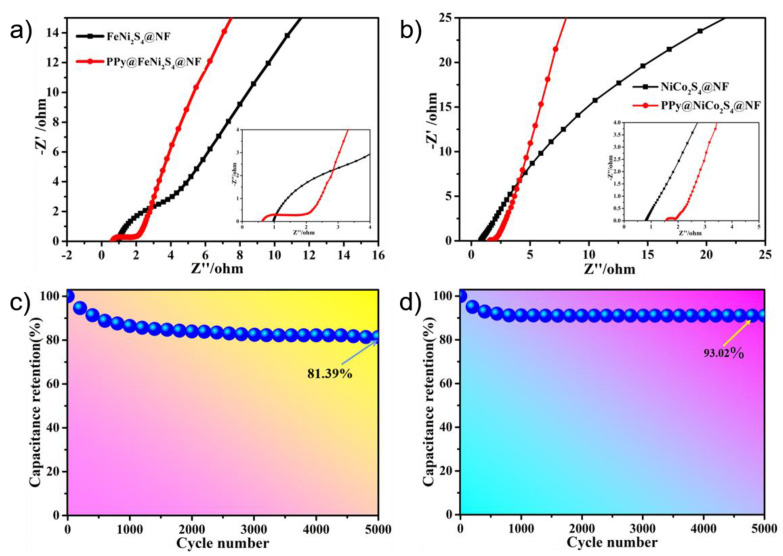
(**a**) Nyquist plots of PPy@FeNi_2_S_4_@NF and FeNi_2_S_4_@NF electrodes. (**b**) Nyquist plots of PPy@NiCo_2_S_4_@NF and NiCo_2_S_4_@NF electrode. (**c**,**d**) Cycling performance of PPy@FeNi_2_S_4_@NF and PPy@NiCo_2_S_4_@NF at a current density of 10 A·g^−1^.

The electrochemical impedance spectroscopy (EIS) curve consists of two parts, which represent charge transfer resistance and ion diffusion resistance [55,56]. According to the semicircle radius of the curve in the high-frequency region, we can see that in Figure 5a, the resistance of the charge transfer resistance of FeNi_2_S_4_@NF-6 is about 2.7 Ω, and that of the charge transfer resistance of PPy@FeNi_2_S_4_@NF-6 is about 1.4 Ω, which indicates that it has much lower diffusion resistance and faster ion diffusion. In the low-frequency region, the slope of the straight line becomes the reference standard. The larger the slope, the shorter the electrolyte–ion diffusion path [57,58]. Furthermore, Figure 5b shows that the resistance value of the charge transfer resistance of NiCo_2_S_4_@NF-6 is 0.4 Ω, and the resistance value of the charge transfer resistance of PPy@NiCo_2_S_4_@NF-6 is 0.3 Ω, indicating that it has the ability of rapid charge transfer. The overall comparison shows that the PPy@FeNi_2_S_4_@NF-6 and PPy@NiCo_2_S_4_@NF electrode material has lower resistance and excellent electrochemical performance, which is attributed to their combination with carbon-based materials.

The excellent cycling performance of the device is a fundamental feature. Figure 5c shows a graph of the cycle stability of the PPy@FeNi_2_S_4_@NF-6 electrode material. After 5000 cycles at 10 A·g^−1^, the final specific capacitance is 3181.1 F·g^−1^, which is 81.39% of the initial specific capacitance. Moreover, Figure 5d shows the cycle stability curve of PPy@NiCo_2_S_4_@NF-6 after 5000 cycles at 10 A·g^−1^ (specific capacitance is 4783.83 F·g^−1^), which is 93.02% of the initial specific capacitance. The overall comparison shows that the resistance of the PPy@FeNi_2_S_4_@NF-6 and Ppy@NiCo_2_S_4_@NF-6 electrode material is small, which is attributed to the composite with a specific composition and core–shell structure and which provides excellent conductivity for the electrode material [59].

**Figure 6:**
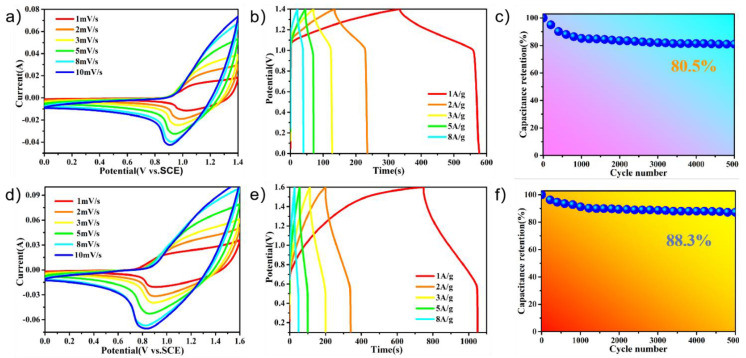
(**a**) CV curves of PPy@FeNi_2_S_4_@NF-6//AC asymmetric supercapacitor at various sweeping rates. (**b**) GCD curves of PPy@FeNi_2_S_4_@NF-6//AC asymmetric supercapacitor at various current densities. (**c**) Cycling performance of PPy@FeNi_2_S_4_@NF//AC at a current density of 10 A·g^−1^. (**d**) CV curves of PPy@NiCo_2_S_4_@NF-6//AC asymmetric supercapacitor at various sweeping rates. (**e**) GCD curves of PPy@NiCo_2_S_4_@NF-6//AC asymmetric supercapacitor at various current densities. (**f**) Cycling performance of PPy@NiCo_2_S_4_@NF//AC at a current density of 10 A·g^−1^.

Figure 6a shows a CV curve of PPy@FeNi_2_S_4_@NF-6//AC at different scan rates, and Figure S8 shows the CV curves of the PPy@FeNi_2_S_4_@NF-6//AC asymmetric supercapacitor (ASC) at different voltage windows, from which the stable operating voltage of the device can be extended up to 1.6 V, as expected. Then, we chose 0–1.4 V as the optimal working potential window of this asymmetric supercapacitor for further investigation. Figure 6a shows a CV curve of PPy@FeNi_2_S_4_@NF-6//AC at different scan rates. The positively charged ions were adsorbed onto the surface of the activated carbon through electrostatic action, and the negatively charged ions were formed through an oxidation reaction in the PPy@FeNi_2_S_4_@NF-6 composite material [60]. A potential difference was formed between the two electrodes to store the charge. Figure 6b shows that the specific capacity of 173.3 F·g^−1^ at 1 A·g^−1^ and the assembled capacitor have an energy density of 47.2 Wh·kg^−1^ and a power density of 699.9 W·kg^−1^. Furthermore, regularly triangular shapes of the GCD curves suggest the ideal capacitance performance and reversible behaviors of the electrode. In Figure 6c, the supercapacitor has about 80.5% of the initial capacitance retained after 5000 cycles at 10 A·g^−1^. In addition, the CV curve of PPy@NiCo_2_S_4_@NF-6//AC at different scan rates is shown in Figure 6d, and the CV curves of the PPy@NiCo_2_S_4_@NF-6//AC asymmetric supercapacitor at different voltage windows are shown in Figure S9. With the scanning rate increasing, there is no obvious distortion in the CV curve, which indicates that the asymmetric supercapacitor has an instantaneous current response and good capacitance behavior [46]. At a current density of 1 A·g^−1^, PPy@FeNi_2_S_4_@NF-6//AC has a specific capacity of 188.75 F·g^−1^, as shown in Figure 6e. It also demonstrates an excellent electrochemical performance with an energy density of 67.11 Wh·kg^−1^ and a power density of 800 W·kg^−1^. We can see in Figure 6f that the supercapacitor composed by us has a high capacitance retention rate of 88.3% after 5000 cycles at 10 A·g^−1^. Figure S10 and Table S1 (Supplementary Materials) show the Ragone plot relating the energy density to the power density of the ASC devices. The results show that the PPy@FeNi_2_S_4_@NF-6//AC and PPy@NiCo_2_S_4_@NF-6//AC devices have great practical application potential.

According to the semicircle radius of the curve in the high-frequency region, we can see that in Figure S11, the resistance of the charge transfer resistance of PPy@FeNi_2_S_4_@NF-6//AC and PPy@NiCo_2_S_4_@NF-6//AC are about 1.5 and 1.8 Ω. In the low-frequency region, the linear part of the signal is closer to the imaginary axis, which indicates that its diffusion resistance is much lower, and the ion diffusion speed of the electrolyte is much faster. In the high-frequency region, the intercept between the impedance arc and real axis is low, and the electrode has low combined resistance and realizes low inherent resistance of the electrode material, which is of great benefit to the excellent capacitance behavior of the electrode [61].

## 3. Experimental Section

### 3.1. Materials

All the chemical reagents in this experiment were of analytical purity and were directly used without any further purification.

### 3.2. Preparation of Materials

Synthesis of FeNi_2_S_4_@NF: 0.207 g of Ni(NO_3_)_2_·6H_2_O and 0.577 g of Fe(NO_3_) _2_·9H_2_O were added with 40 mL of deionized water and stirred. After stirring for 5 min, 0.216 g of CO(NH_2_)_2_ and 0.135 g NH_4_F were added for further stirring for 20 min and ultrasonic for 10 min. Then, the reaction was carried out in a high-pressure reactor at 120 °C into a 100 mL Teflon-lined autoclave, and the materials were synthesized at different reaction times (6, 8 and 10 h). After the reaction was completed, the reaction product was washed with ethanol and deionized water to remove the remaining impurities. Nickel foam was dried at 60 °C for 12 h to obtain iron–nickel double hydroxide based on nickel foam. According to a load of the precursor, the dosage of C_2_H_5_NS needed was calculated, and the ratio was 1:4. A corresponding amount of C_2_H_5_NS was weighed and dissolved in 30 mL deionized water, stirred with the precursor for 20 min, and then reacted at 120 °C in a Teflon-lined autoclave for 5 h; then, the product was rinsed with ethanol and deionized water to wash off the remaining impurities, and the nickel foam was dried at 60 °C for 12 h to obtain FeNi2S4@NF-x (x = 6, 8, 10 h).

Synthesis of PPy@FeNi_2_S_4_@NF: 0.213 g P-TSA was put into a 50 mL clean beaker, 30 mL of anhydrous ethanol was added, and stirred for 5 min in a water bath at 0~5 °C. With the medicine completely dissolved, add 0.2 mL Py, 0.24 g APS and 20 mL deionized water to the solution, and stir vigorously for 30 s, then loaded in the dark for 4 h. Finally, the samples were washed with deionized water and ethanol.

Synthesis of NiCo_2_S_4_@NF: 0.29g Ni(NO_3_)_2_·6H_2_O and 0.58g Co(NO_3_)_2_·6H_2_O, were added to and stirred in 80 mL deionized water; then, 0.72 g CO(NH_2_)_2_ was added and continuously stirred for 20 min. Then, treated nickel foam was placed into the above solution and ultrasonic for 10 min. Then, a Teflon-lined autoclave was used to react at 120 °C, and materials were synthesized at different reaction times. The reaction product was washed with ethanol and deionized water to remove the remaining impurities. The vulcanization process is the same as the above FeNi_2_S_4_@NF materials.

Synthesis of PPy@NiCo_2_S_4_@NF: This process is the same as the above PPy@FeNi_2_S_4_@NF materials.

### 3.3. Materials Characterization

Phase information of the composites was tested by a powder X-ray diffractometer (XRD; Rigaku, TTR-Ⅲ). The morphology and microstructure of the composites were observed by scanning electron microscope (FE-SEM, Hitachi, SU8000) and high-resolution transmission electron microscope (HRTEM, JEM-2010). Valence analysis of the samples was conducted by X-ray photoelectron spectroscopy (XPS, Thermo Scientific ESCALAB 250Xi system).

### 3.4. Electrochemical Characterization

Electrochemical workstation (CHI 760E) was used to observe the electrochemical performance of the PPy@FeNi_2_S_4_@NF and PPy@NiCo_2_S_4_@NF electrodes in a three-electrode installation. In this test, platinum was used as a counter electrode, Ag/AgCl as a reference electrode PPy@FeNi_2_S_4_@NF and PPy@NiCo_2_S_4_@NF as a working electrode, with a solution of 1 M KOH as electrolyte. Cyclic voltammetry (CV) was performed between −0.8 and 0.8 V at various scan rates (from 1 to 10 mV·s^−1^). Charge transfer resistances were measured in a voltage window from 0 to 0.35 V at various current densities. EIS measurements were carried out with a 5 mV sinusoidal voltage in frequencies from 100 kHz to 0.01 Hz. The specific capacitances (C, F g^−1^), energy density (E, Wh kg^−1^) and power density (P, W kg^−1^) of the samples were calculated by Equations (1)–(3) based on GCD plots [28,29,30]:

C = (I × Δt)/m × ΔV (1)

E = C × (ΔV)^2^/2 × 3.6 (2)

P = E/t (3)

where Δt (s), I (A), m (g), ΔV (V) and t (h) designate the discharge times, currents, the mass of the electrode, and the applied potential, respectively.

## 4. Conclusions

In conclusion, the core–shell structure of PPy@FeNi_2_S_4_@NF and PPy@NiCo_2_S_4_@NF were prepared by in situ self-assembly to solve the instability and low electron and ion transports toward a high-performance supercapacitor. The PPy@FeNi_2_S_4_@NF and PPy@NiCo_2_S_4_@NF hybrid materials showed higher specific capacitances of 3870.3 and 5771.4 F·g^−1^ at a current density of 1 A·g^−1^, as well as remarkable cycling stability (about 81.39% and 93.02% after 5000 cycles). Furthermore, the PPy@FeNi_2_S_4_@NF-6//AC and PPy@NiCo_2_S_4_@NF-6//AC asymmetric supercapacitor demonstrate high-energy density of 47.2 Wh·kg^−1^ at a power density of 699.9 W·kg^−1^ and 67.11 Wh·kg^−1^ at a power density of 800 W·kg^−1^. This work indicates the great potential of an internal/external bicontinuous conductive network design in new-fashioned energy storage applications.

## Data Availability

Not applicable.

## Supplementary Materials

The following supporting information can be downloaded at: https://www.mdpi.com/article/10.3390/molecules27238168/s1, Figure S1: SEM images of PPy@FeNi2S4@NF composites at various reaction times; Figure S2: SEM images of PPy@NiCo2S4@NF composites at various reaction times; Figure S3: XPS survey scan of PPy@FeNi2S4@NF and PPy@NiCo2S4@NF; Figure S4: CV curves of the FeNi2S4@NF electrode at various sweeping rates. CV contrast curves of the FeNi2S4@NF and PPy@FeNi2S4@NF electrode; Figure S5: Specific capacitance of FeNi2S4@NF electrode at different current densities; Figure S6: Specific capacitance of NiCo2S4@NF electrode at different current densities; Figure S7: GCD contrast curve of PPy@FeNi2S4@NF and PPy@NiCo2S4@NF at different reaction time; Figure S8: CV curves of PPy@FeNi2S4@NF-6//AC asymmetric supercapacitor at different voltage windows; Figure S9: CV curves of PPy@NiCo2S4@NF-6//AC asymmetric supercapacitor at different voltage window; Figure S10: Ragone plots of energy density and power density of PPy@FeNi2S4@NF-6//AC and PPy@NiCo2S4@NF-6//AC; Figure S11: Nyquist plots of PPy@FeNi2S4@NF-6//AC and PPy@NiCo2S4@NF-6//AC; Table S1: Comparison of the electrochemical performance of the PPy@FeNi2S4@NF and PPy@NiCo2S4@NF electrode with performances of reported electrode [32,59,62,63,64,65,66].

## Sample Availability

Samples of the compounds FeNi2S4@NF, PPy@FeNi2S4@NF, NiCo2S4@NF and PPy@NiCo2S4@NF are available from the authors.
