# Supplementary material for: Design of an Internal/External Bicontinuous Conductive Network for High-Performance Asymmetrical Supercapacitors
Source: Molecules. 2022 Nov 23;27(23):8168. doi: 10.3390/molecules27238168 (PMC9736552; doi:10.3390/molecules27238168)
Supplement: Supplementary file 1 [file molecules-27-08168-s001.zip › molecules-2021331-supplementary.pdf]

Supplementary Material

# Design of an Internal/External Bicontinuous Conductive Network for High-Performance Asymmetrical Supercapacitors

**Anran Shi [1], Xiumei Song [2], Lei Wei [1], Huiyuan Ma [1,*], Haijun Pang [1], Weiwei Li [3], Xiaowei Liu [3] and Lichao Tan [1,2,3,4,*]**

[1] School of Materials Science and Chemical Engineering, Harbin University of Science and Technology, Harbin 150040, China
[2] Institute of Carbon Neutrality, Zhejiang Wanli University, Ningbo 315100, China
[3] Chilwee Power Co., Ltd., No. 18 Chengnan Road, Huaxi Industrial Zone, Changxing 313100, China
[4] State Key Laboratory of Clean Energy Utilization, School of Materials Science and Engineering, Zhejiang University, Hangzhou 310027, China
* Correspondence: mahy017@163.com (H.M.); tanlcking@163.com (L.T.)

**Table of Contents**

1. SEM images of PPy@$FeNi_2S_4$@NF composites at various reaction times.
2. SEM images of PPy@$NiCo_2S_4$@NF composites at various reaction times.
3. XPS survey scan of the PPy@$FeNi_2S_4$@NF and PPy@$NiCo_2S_4$@NF
4. CV contrast curves of the $FeNi_2S_4$@NF and PPy@$FeNi_2S_4$@NF electrode, CV contrast curves of the $NiCo_2S_4$@NF and PPy@$NiCo_2S_4$@NF electrode
5. Specific capacitance of $FeNi_2S_4$@NF electrode at different current densities.
6. Specific capacitance of $NiCo_2S_4$@NF electrode at different current densities.
7. GCD contrast curve of PPy@$FeNi_2S_4$@NF and PPy@$NiCo_2S_4$@NF at different reaction time.
8. CV curves of PPy@$FeNi_2S_4$@NF-6//AC asymmetric supercapacitor at different voltage window.
9. CV curves of PPy@$NiCo_2S_4$@NF-6//AC asymmetric supercapacitor at different voltage window
10. Ragone plots of energy density and power density of PPy@$FeNi_2S_4$@NF-6//AC and PPy@$NiCo_2S_4$@NF-6//AC.
11. Nyquist plots of PPy@$FeNi_2S_4$@NF-6//AC and PPy@$NiCo_2S_4$@NF-6//AC.

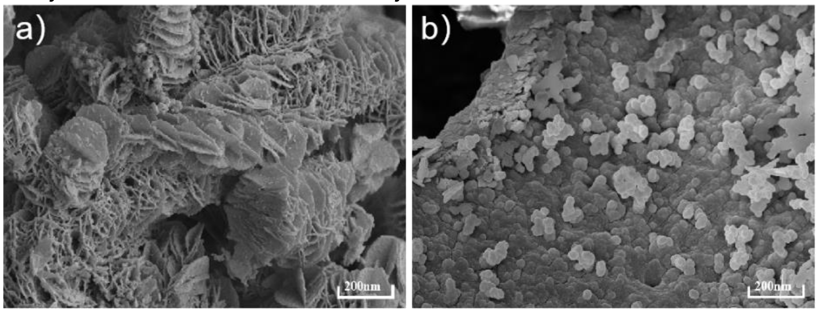


**Figure S1.** (**a,b**) SEM images of PPy@$FeNi_2S_4$@NF composites at 6, 10 h reaction times.

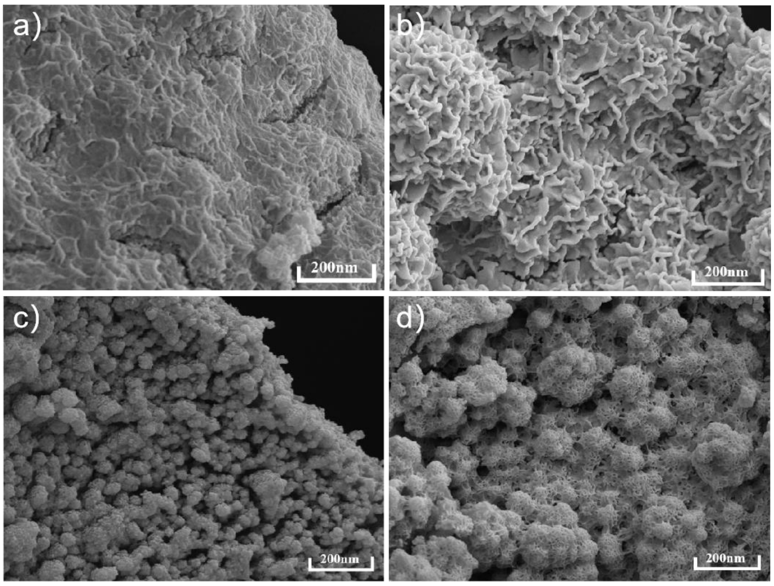


**Figure S2.** (**a–d**) SEM images of PPy@$NiCo_2S_4$@NF composites at 4, 8, 10, 12 h reaction times.

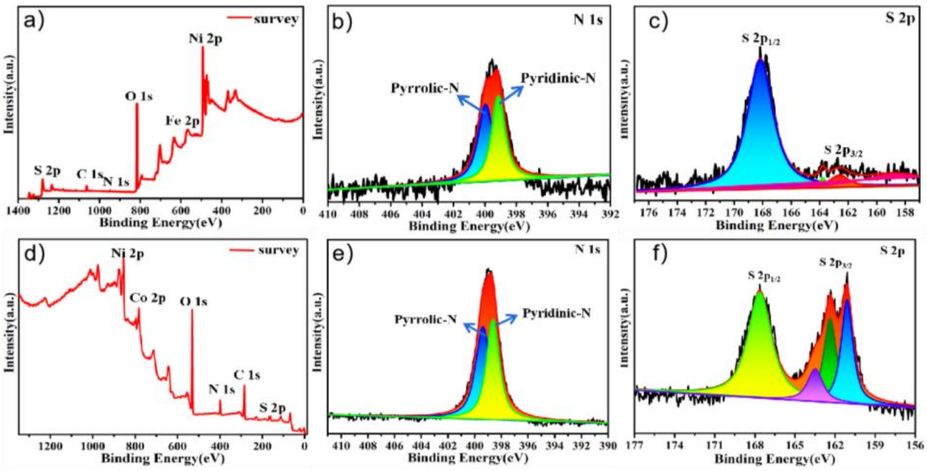


**Figure S3.** XPS survey scan of the PPy@$FeNi_2S_4$@NF, high resolution XPS spectra of (**a**) full spectrum, (**b**) N 1s and (**c**) S 2p, XPS survey scan of the PPy@$NiCo_2S_4$@NF, high resolution XPS spectra of (**d**) full spectrum, (**e**) N 1s and (f) S 2p.

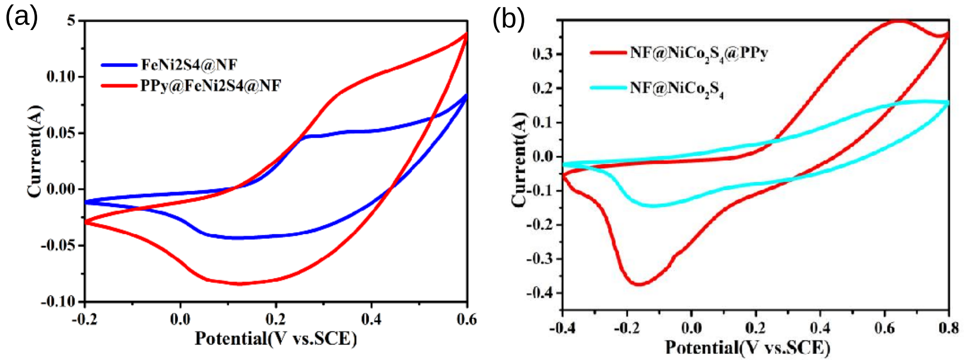


**Figure S4.** (**a**) CV contrast curves of the $FeNi_2S_4$@NF and PPy@$FeNi_2S_4$@NF electrode, (**b**) CV contrast curves of theN-iCo$_2$S$_4$@NF and PPy@NiCo$_2$S$_4$@NF electrode

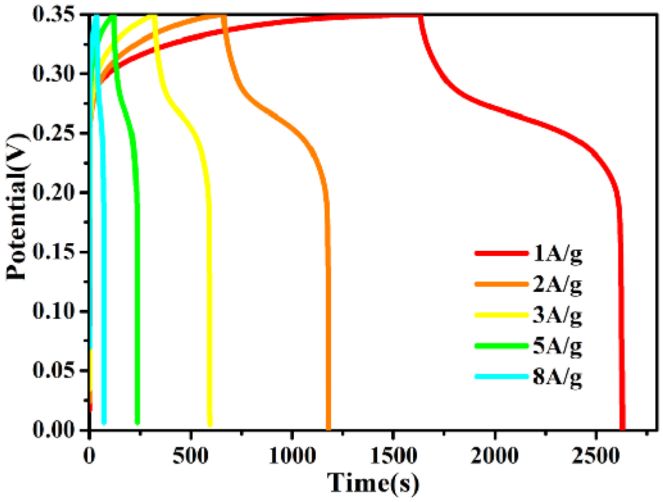


**Figure S5.** Specific capacitance of $FeNi_2S_4$@NF electrode at different current densities.

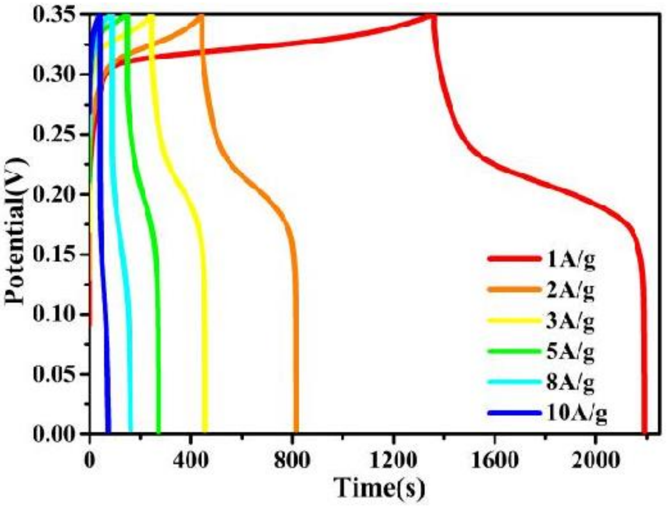


**Figure S6.** Specific capacitance of $NiCo_2S_4$@NF electrode at different current densities.

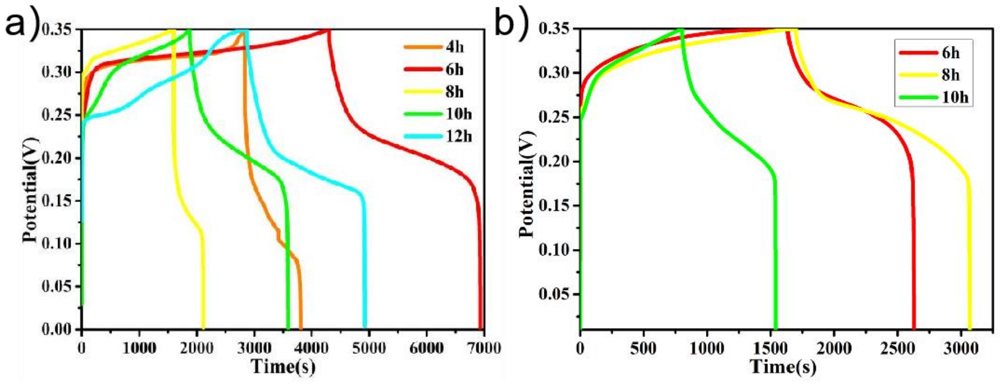


**Figure S7.** GCD contrast curve of (**a**) PPy@$FeNi_2S_4$@NF and (**b**) PPy@$NiCo_2S_4$@NF at different reaction time.

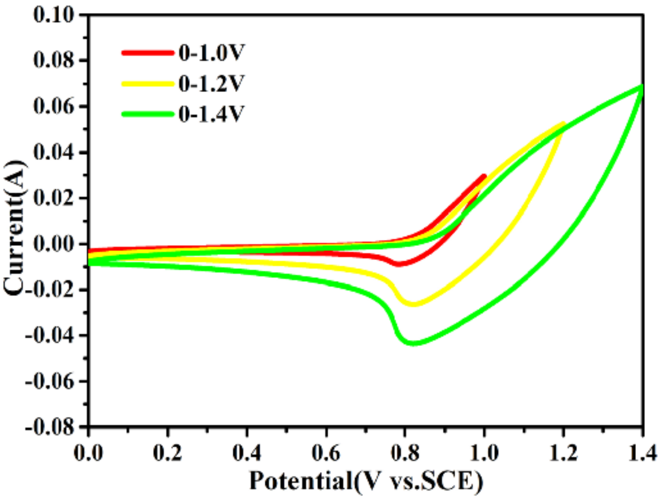


**Figure S8.** CV curves of PPy@$FeNi_2S_4$@NF-6//AC asymmetric supercapacitor at different voltage window.

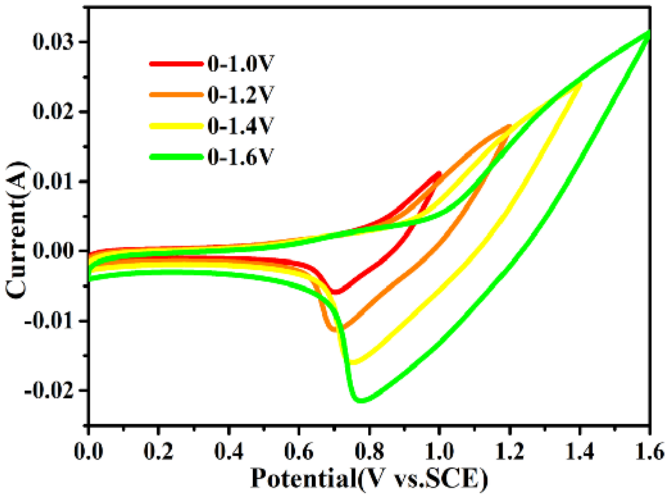


**Figure S9.** CV curves of PPy@$NiCo_2S_4$@NF-6//AC asymmetric supercapacitor at different voltage window.

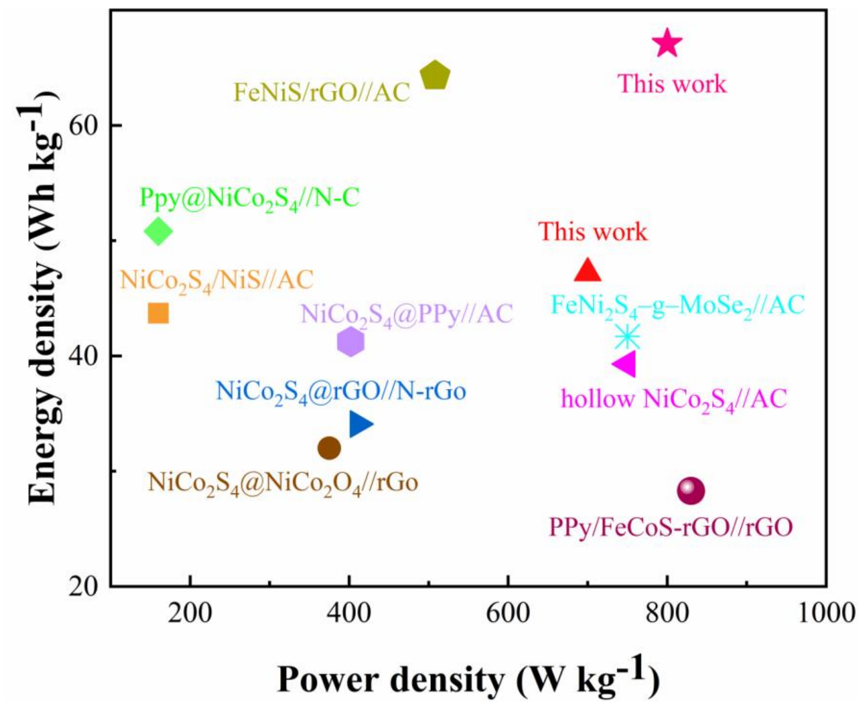


**Figure S10.** Ragone plots of energy density and power density of PPy@$FeNi_2S_4$@NF//AC and PPy@$NiCo_2S_4$@NF//AC.

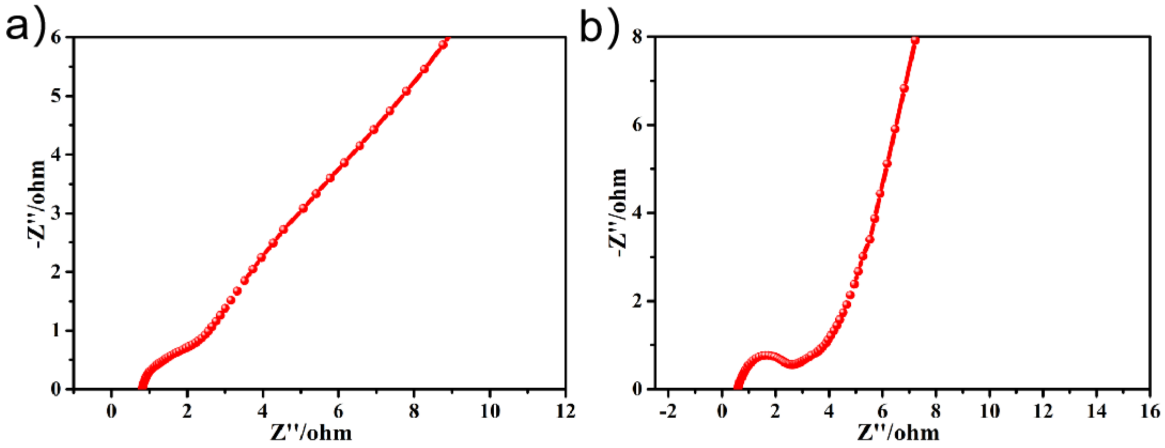


**Figure S11.** Nyquist plots of (**a**) PPy@$FeNi_2S_4$@NF-6//AC and (**b**) PPy@$NiCo_2S_4$@NF-6//AC.

**Table S1.** Comparison of the electrochemical performance of the PPy@$FeNi_2S_4$@NF and PPy@$NiCo_2S_4$@NF electrode with performances of reported electrode.

| Electrode material | Energy density | Power density | Potential (V) | Ref |
|---|---|---|---|---|
| **PPy@$FeNi_2S_4$@NF//AC** | 47.2 Wh/kg | 699.9 W/kg | 0–1.6 | This work |
| **PPy@$NiCo_2S_4$@NF//AC** | 67.1 Wh/kg | 800 W/kg | 0–1.6 | This work |
| **Ppy@$NiCo_2S_4$//N-C** | 50.8 Wh/kg | 160 W/kg | 0–1.6 | [1] |
| **$NiCo_2S_4$@PPy//AC** | 41.2 Wh/kg | 402.1 W/kg | 0–1.6 | [2] |
| **PPy/FeCoS-rGO//rGO** | 28.3 Wh/kg | 830 W/kg | 0–1.4 | [3] |
| **hollow $NiCo_2S_4$//AC** | 39.3 Wh/kg | 749.6 W/kg | 0–1.5 | [4] |
| **$NiCo_2S_4$@rGO//N-rGo** | 34.1 Wh/kg | 411 W/kg | 0–1.6 | [5] |
| **FeNiS/rGO//AC** | 64.3 Wh/kg | 508 W/kg | 1–3 | [6] |
| **$NiCo_2S_4$/NiS//AC** | 43.7 Wh/kg | 160 W/kg | 0–1.6 | [7] |
| **$NiCo_2S_4$@$NiCo_2O_4$//rGo** | 32.0 Wh/kg | 375 W/kg | 1–1.5 | [8] |

**References:**


1. Zheng, Y.; Xu, J.; Yang, X.; Zhang, Y.; Shang, Y.; Hu, X., Decoration NiCo 2 S 4 nanoflakes onto Ppy nanotubes as core-shell heterostructure material for high-performance asymmetric supercapacitor. *Chemical Engineering Journal* **2017**, 111-121.
2. Li, J.; Zou, Y.; Li, B.; Xu, F.; Xiang, C., Polypyrrole-wrapped NiCo2S4 nanoneedles as an electrode material for supercapacitor applications. *Ceramics International* **2021**.
3. Karimi, A.; Kazeminezhad, I.; Naderi, L.; Shahrokhian, S., Construction of a Ternary Nanocomposite, Polypyrrole/Fe–Co Sulfide-Reduced Graphene Oxide/Nickel Foam, as a Novel Binder-Free Electrode for High-Performance Asymmetric Supercapacitors. *The Journal of Physical Chemistry C* **2020,** XXXX, (XXX).
4. Shi, Z.; Shen, X.; Zhang, Z.; Wang, X.; Gao, N.; Xu, Z.; Chen, X.; Liu, X., Hierarchically urchin-like hollow Ni-Co 2 S 4 prepared by a facile template-free method for high-performance supercapacitors. *Journal of Colloid and Interface Science* **2021**.
5. Zhao, Y.; Zhang, H.; Lin, Y.; Chen, J.; Cheng, A., Design and construction of nickel-cobalt-sulfide nanoparticles in-situ grown on graphene with enhanced performance for asymmetric supercapacitors. *Diamond and Related Materials* **2020,** 108.
6. Li, L.; Zhang, L.; Cheng, B.; You, W.; Yu], J., 0D/2D (Fe0.5Ni0.5)S2/rGO nanocomposite with enhanced supercapacitor and lithium ion battery performance. *Journal of Power Sources* **2019**.
7. Xu, R.; Lin, J.; Wu, J.; Huang, M.; Fan, L.; He, X.; Wang, Y.; Xu, Z., A two-step hydrothermal synthesis approach to synthesize NiCo2S4/NiS hollow nanospheres for high-performance asymmetric supercapacitors. *Applied Surface Science* **2017,** 422, (nov.15), 597-606.
8. Singh, A.; Ojha, S. K.; Singh, M.; Ojha, A. K., Controlled synthesis of NiCo2S4@NiCo2O4 Core@Shell nanostructured arrays decorated over the rGO sheets for high-performance asymmetric supercapacitor. *Electrochimica Acta* **2020,** 349, 136349.
